# Agreement between two real-time commercial PCR kits and an in-house real-time PCR for diagnosis of mucormycosis

**DOI:** 10.1128/spectrum.03585-23

**Published:** 2024-06-25

**Authors:** Lovanirina Clémencia Rafanomezantsoa, Estelle Sabourin, Nadia Guennouni Sebbouh, Emilie Sitterlé, Nada Ben Halima, Yannick Sonjah Raveloarisaona, Gilles Quesne, Eric Dannaoui, Marie-Elisabeth Bougnoux

**Affiliations:** 1 Université Paris Cité, Faculté de Médecine, APHP, Hôpital Necker Enfants-Malades, Hôpital Européen Georges Pompidou, Unité de Parasitologie-Mycologie, Service de Microbiologie, Paris, France; 2 Dynamyc Research Group, Paris Est Créteil University (UPEC, EnvA), Paris, France; 3 Biologie et Pathogénicité fongiques, Département de Mycologie, Institut Pasteur, Paris, France; Jawaharlal Nehru Centre for Advanced Scientific Research, Bangalore, India

**Keywords:** mucormycosis, PCR, molecular diagnosis, diagnosis, MycoGENIE *Aspergillus-Mucorales* spp. Real-Time PCR Kit, Fungiplex Kit Mucorales Real-Time PCR Kit

## Abstract

Mucormycosis is a severe and emerging invasive fungal infection associated with high mortality rates. Early diagnosis is crucial for initiating specific antifungal treatment, with molecular tools currently representing the most efficient diagnostic approach. Presently, a standardized in-house real-time PCR method is widely employed for diagnosing mucormycosis. Our study aimed to evaluate the agreement for the Mucorales DNA detection between two commercial real-time PCR assays—the Fungiplex Mucorales Real-Time PCR Kit and the MycoGENIE *Aspergillus-Mucorales* spp. Real-Time PCR Kit—in comparison with the in-house PCR. We retrospectively analyzed 58 samples previously identified as positive for Mucorales using the in-house PCR. These samples, obtained from 22 patients with proven or probable mucormycosis, were tested with both commercial kits. Additionally, samples from 40 patients without mucormycosis served as negative controls. Our findings revealed that the MycoGENIE Kit demonstrated superior performance in detecting Mucorales DNA in samples identified as positive by the in-house PCR. Notably, we observed minimal variability in cycle threshold (CT) values when comparing the results of the MycoGENIE Kit with those of the in-house PCR, with an average difference of 1.8 cycles. In contrast, the Fungiplex Kit exhibited a larger discrepancy in CT values compared to the in-house PCR, with an average difference of 4.1 cycles. The MycoGENIE Kit exhibited very good agreement (kappa of 0.82) with the in-house PCR for detecting Mucorales DNA across various sample types. These findings are important for the choice of kits that could be used to diagnose mucormycosis in clinical microbiology laboratories.

**IMPORTANCE:**

Early diagnosis of mucormycosis is crucial for initiating effective treatment. The detection of Mucorales DNA by PCR in serum has revolutionized the diagnosis of this infection. However, the use of in-house methods can be time consuming. The availability of a commercial kit eliminates the need for in-house assay development, reducing laboratory workload and ensuring consistent performance across different healthcare settings. Currently, there are several commercial assays available, but many have limited evaluation. In this study, we compared two commercial kits and found that the MycoGENIE Kit offers a promising alternative to the in-house method.

## INTRODUCTION

Invasive fungal infections continue to pose a growing challenge in modern medicine (1, 2). Bone marrow transplantation in patients with hematological malignancies and solid organ transplantation with corticotherapy promote immunosuppression, which is the main risk factor for the development of invasive fungal infections (3–5). Among these, mucormycosis stands out as a severe and often fatal invasive fungal infection, with high mortality rate of >40% (6). The prognosis of the patient depends on the precocity of the diagnosis of the mucormycosis in order to initiate the specific treatment with liposomal amphotericin B and surgery (7, 8). While mycological work-up (including direct examination and culture) and histology remain the gold-standard diagnostic methods according to the recommendations of the EORTC/MSGERC (European Organization for the Research and Treatment of Cancer/Mycoses Study Group Education and Research Consortium), their results may be delayed (9). Moreover, biopsies are not always available depending on the site of infection and the clinical condition of the patient (10). Recently, in-house real-time PCR assays have emerged as promising alternatives, demonstrating reliability in early detection (11). Notably, PCR in serum can be positive several days before the conventional diagnosis (12), making it a valuable screening tool for at-risk patients suspected of mucormycosis. Moreover, PCR has prognostic value and can be used to monitor the antifungal treatment efficacy (11).

Currently, three commercial kits are available for detecting Mucorales DNA in serum, respiratory samples, and biopsies (MucorGenius by PathoNostics, MycoGENIE Mucorales by Ademtech, and Fungiplex Mucorales by Bruker). MucorGenius is the kit that has been the first to be evaluated (13–19). Furthermore, the MycoGENIE Kit was recently prospectively evaluated as a molecular workflow for the diagnosis of both mucormycosis and aspergillosis in sera from high-risk patients (20). Currently, the in-house Mucorales real-time PCR developed by Millon et al. (18, 21) has been standardized and evaluated and is adopted by numerous laboratories. Nevertheless, for routine laboratories, the availability of commercial kits is of importance as in-house PCR is time consuming and labor intensive. Our study aims to evaluate the agreement between the results obtained with the MycoGENIE Mucorales and Fungiplex Mucorales Kits and those of in-house PCR, within our center.

## MATERIALS AND METHODS

### Patients and samples

We identified 22 patients for whom the diagnosis of proven or probable mucormycosis according to the EORTC criteria (22) was done between 01 January 2017 and 29 March 2022. For these 22 patients, 58 samples, positive (CT <40) by using Mucorales PCR (called in-house real-time PCR) developed and evaluated by Millon et al. (21), were available at the Parasitology-Mycology Laboratory of Necker Children’s Hospital, Paris. The 58 samples originated from blood (35, 60.3%), skin biopsies (14, 24.1%), respiratory samples (2, 3.4%), sinus lavage fluids (2, 3.4%), cerebrospinal fluids (2, 3.4%), and other types of samples (1 lung biopsy, 1 biopsy of the pleura, and 1 biopsy of the diaphragmatic false membrane). Among the 58 samples, 25 samples (43.1%) were positive for *Mucor/Rhizopus,* 22 (37.9%) for *Lichtheimia,* and 11 (19%) for *Rhizomucor*, with cycle thresholds ranging from 22 to 39. The underlying disease, the site of infection, and the origin of samples for the 22 patients with mucormycosis are presented in Table 1.

**TABLE 1:** Characteristics of patients and samples^*a*^

Patient	Underlying disease	Site of infection	Sample type
1	Myelodysplastic syndrome	Lung	Serum
2	Silicosis, influenza	Lung	Serum
3	Wegener’s disease, rituximab	Lung	Serum
4	HSCT, GVH, sickle cell disease, diabetes	Lung, brain	Serum
5	Lung transplant	Sinus	Serum
6	Lung transplant	Sinus	Sinus lavage fluid
7	KID syndrome	Cutaneous	Skin biopsy
8	AML	ORL (eye, sinus)	Serum
9	Mediastinitis	Lung, disseminated	Biopsy of the pleura and biopsy of the diaphragmatic false membrane
10	HSCT, skin nodule	Lung, cutaneous	Serum and lung biopsy
11	Diabetes, COVID-19	Brain, sinus maxillary	Cerebrospinal fluid
12	Large cell lymphoma	Lung	Serum
13	Refractory acute leukemia	Lung	Serum
14	NA	NA	Serum
15	HSCT, ALL	Lung	Serum
16	AML	Disseminated (brain, skin, ENT)	Serum
17	Myeloma	Lung	Serum
18	AML	Lung	Serum and BAL
19	Lung cancer	Lung	Serum
20	Hematological malignancy	NA	Serum
21	HSCT	Lung	Serum
22	CGD, HSCT	Lung	Serum and expectoration

^*a*^ HSCT, hematopoietic stem cell transplant; GVH, graft versus host disease; AML, acute myeloid leukemia; KID syndrome, keratitis ichthyosis deafness syndrome; ALL, acute lymphoid leukemia; ENT, ear nose throat; BAL, bronchoalveolar lavage; CGD, chronic granulomatous disease; NA, not available.

A total of 40 samples from 40 different patients without mucormycosis collected in 2022 were selected to serve as negative controls for the two *Mucorales* PCR kits. Among these 40 patients, 20 (20 samples) had a negative result with both in-house *Mucorales* PCR and specific *Aspergillus fumigatus* PCR, and 20 other patients (20 samples) had a negative result by *Mucorales* in-house PCR but had a positive result by specific *Aspergillus fumigatus* PCR assay. As aspergillosis is the most frequent filamentous fungal infection, we have included 20 samples that were positive for *Aspergillus* to ensure that Mucorales DNA detection was not falsely detected in these samples. The samples included 18 respiratory samples, 18 blood samples, 1 cerebrospinal fluid (CSF), and 3 other types of samples.

### Positive control strains

Seven strains of *Mucorales* belonging to six species cultured on Sabouraud supplemented with antibiotics (chloramphenicol and gentamicin) incubated at +27°C were used as positive controls for *Mucorales* PCR: one *Cunninghamella bertholletiae*, one *Rhizopus arrhizus*, two *Rhizomucor pusillus*, one *Mucor circinelloides*, one *Lichtheimia corymbifera,* and one *Mucor irregularis* (formerly *Rhizomucor variabilis*).

### Processing of samples and DNA

For biopsies and other purulent or viscous samples, a pretreatment was performed using a MagNA Lyser apparatus (Roche Diagnostics, Meylan, France).

One milliliter of serum, respiratory samples, or biopsies and 5.0 µL of internal control were extracted using an EMAG automatic device (bioMérieux, Marcy l’Etoile, France); 50 µL of DNA eluates was obtained for serum and 100 µL for respiratory samples and other types of samples.

### Mucorales strains and DNA extraction

DNA extraction from Mucorales strains was performed manually using the MasterPure Yeast DNA Purification Kit (Lucigen, LGC, Biosearch Technologies, USA), according to manufacturer’s instructions, and DNA pellet was resuspended in 100 µL of sterile distilled water.

### Real-time Mucorales PCR assays

The in-house Mucorales real-time PCR assay, described and validated by Millon et al. (12), was performed in routine diagnostic at the Parasitology-Mycology Laboratory of Necker Children’s Hospital, Paris . The PCR assay, targeting the 18S rDNA, included a simple PCR for the detection of *Mucor-Rhizopus* spp. and a multiplex PCR for the detection of the two genera *Lichtheimia* spp. and *Rhizomucor* spp. PCR reactions were performed on CFX96-Biorad apparatus. The CT cut-off value for in-house PCR is <40.

Two real-time PCR commercial kits—Fungiplex *Mucorales* RUO Real-Time PCR Kit (Bruker, Bremen, Germany) and MycoGENIE *Aspergillus-Mucorales* spp. (Ademtech, Pessac, France)—were blinded and evaluated on the same samples that are used for in-house Mucorales PCR, by using DNA eluates kept frozen at −80°C.

Fungiplex (Fungiplex *Mucorales* RUO Real-Time PCR Kit) simultaneously targets *Lichtheimia* spp.*, Syncephalastrum* spp.*, Rhizopus* spp.*, Mucor* spp., *Cunninghamella* spp., *Actinomucor* spp.*, Apophysomyces* spp., *Saksenaea* spp., and *Rhizomucor* spp. Amplification was performed according to the manufacturer’s instructions. Briefly, 5.0 µL of DNA was added to 15 µL of master mix. After incorporation of 1 µL/sample of internal PCR inhibition control included in the kit, amplification was carried out on QuantStudio 5-Applied Biosystems (Thermo Fisher Scientific, Waltham, MA, USA). The thermal cycle conditions included a first denaturation step at +95°C for 15 minutes, followed by 45 cycles of 5 s at +95°C and 30 s at +60°C.

The sensitivity, specificity, and CT thresholds according to the manufacturer are 98.2%, 99.9%, and <45, respectively.

MycoGENIE (MycoGENIE *Aspergillus-Mucorales* spp*.* Real-Time PCR Kit) simultaneously targets rDNA regions of *Rhizomucor pusillus, Mucor indicus, Mucor circinelloides, Mucor plumbeus, Rhizopus arrhizus, Rhizopus stolonifer, Lichtheimia corymbifera, Lichtheimia glauca, Cunninghamella bertholletiae,* and *Mycotypha* spp. Amplification was performed according to the manufacturer’s instructions. Briefly, 15 µL of master mix included the internal PCR inhibition control (2.5 µL/sample) provided in the kit and 10 µL of extract of DNA. The PCR reaction was carried out on QuantStudio 5. The thermal cycle conditions are as follows: a first denaturation step at +95°C for 10 minutes, followed by 45 cycles of 15 s at +95°C and 1 minute at +60°C.

The sensitivity, specificity, CT thresholds, and detection limits according to the manufacturer are 93%, 69%, <45, and 10 gene copies, respectively.

## RESULTS

### Detection of DNA from positive control strains

Among the seven strains belonging to five genera/six species, in-house PCR detected DNA from all species except *C. bertholletiae* and provided accurate identification at the genus level for *Lichtheimia* and *Rhizomucor,* and at the group level for *Mucor/Rhizopus*. The MycoGENIE Kit detected DNA from all the six species, but the kit is not designed to provide identification at the genus/species level. Finally, the Bruker Kit detected DNA from all species except *M. irregularis* (formerly *Rh. variabilis*) and gave an accurate clustering into the three groups of Mucorales identified by the three different fluorochromes.

### Performances of the two commercial kits (Fungiplex and MycoGENIE) in comparison to in-house Mucorales PCR for DNA detection in clinical samples

**Fig 1:**
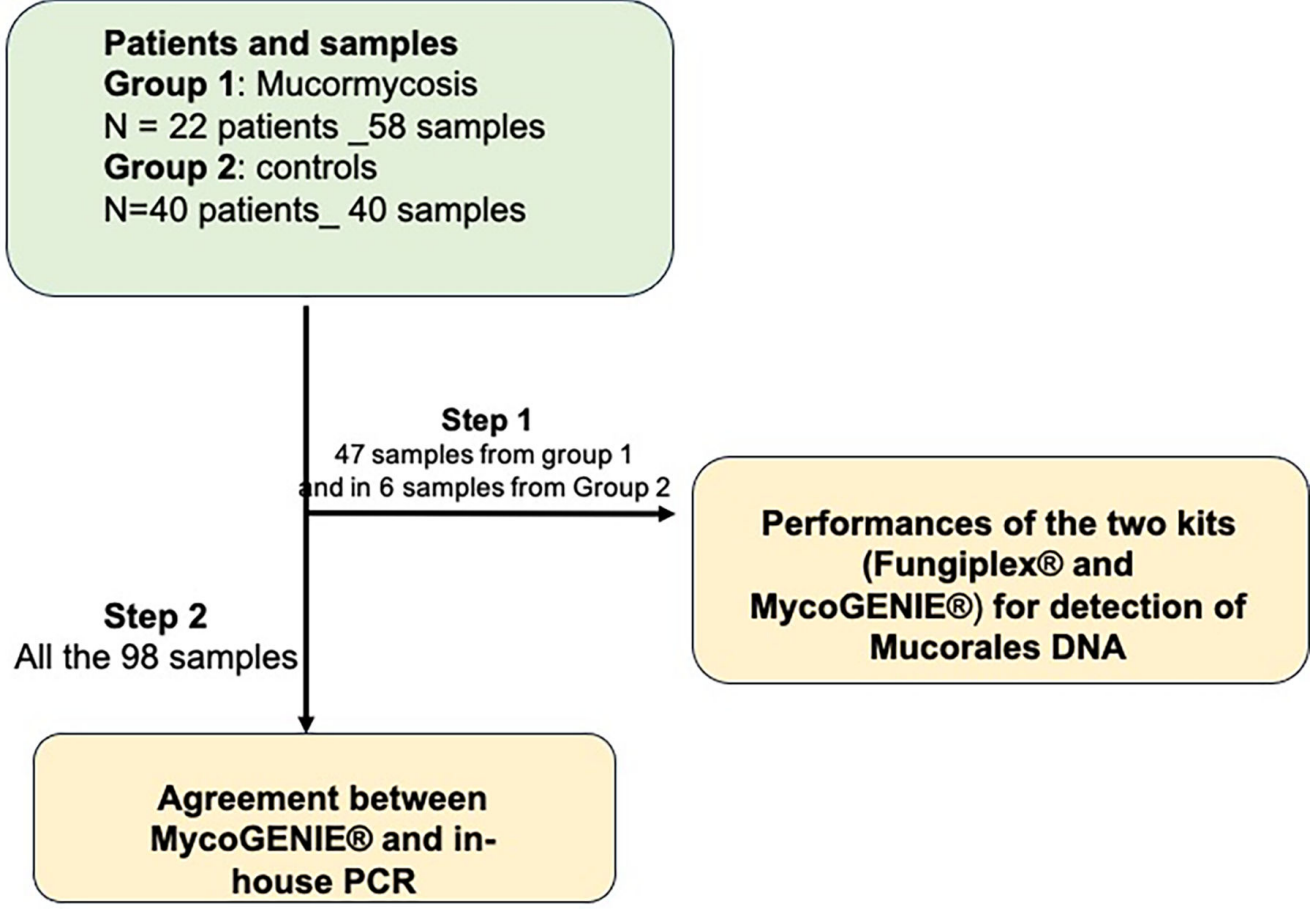
Flow chart of the study. In a first step, 53 samples (47 positive and 6 negative) were used to test the performances of DNA detection by the two commercial kits (Fungiplex and MycoGENIE) in comparison to the in-house PCR. In a second step, all the 98 samples (58 positive and 40 negative) were used for the evaluation of the agreement for DNA detection between the sensitivity and specificity of the MycoGENIE Kit and the in-house PCR.

A total of 53 samples (47 positive samples by in-house Mucorales PCR and 6 negative samples) were used to test the two kits, Fungiplex and MycoGENIE, in parallel (Fig. 1, step 1). Among the 47 positive samples, 39 (83%) were positive by the MycoGENIE (8 false positive), while only 24 (51%) were positive by the Fungiplex Kit (23 false positive) (Table 2). All the six negative samples were also negative with both commercial kits.

**TABLE 2:** PCR results obtained by the two Mucorales kits compared to in-house PCR

Sample	In-house PCR	Fungiplex		MycoGENIE	
		Positive	Negative	Positive	Negative
Positive	47	24	23	39	8
Negative	6	0	6	0	6

**Fig 2:**
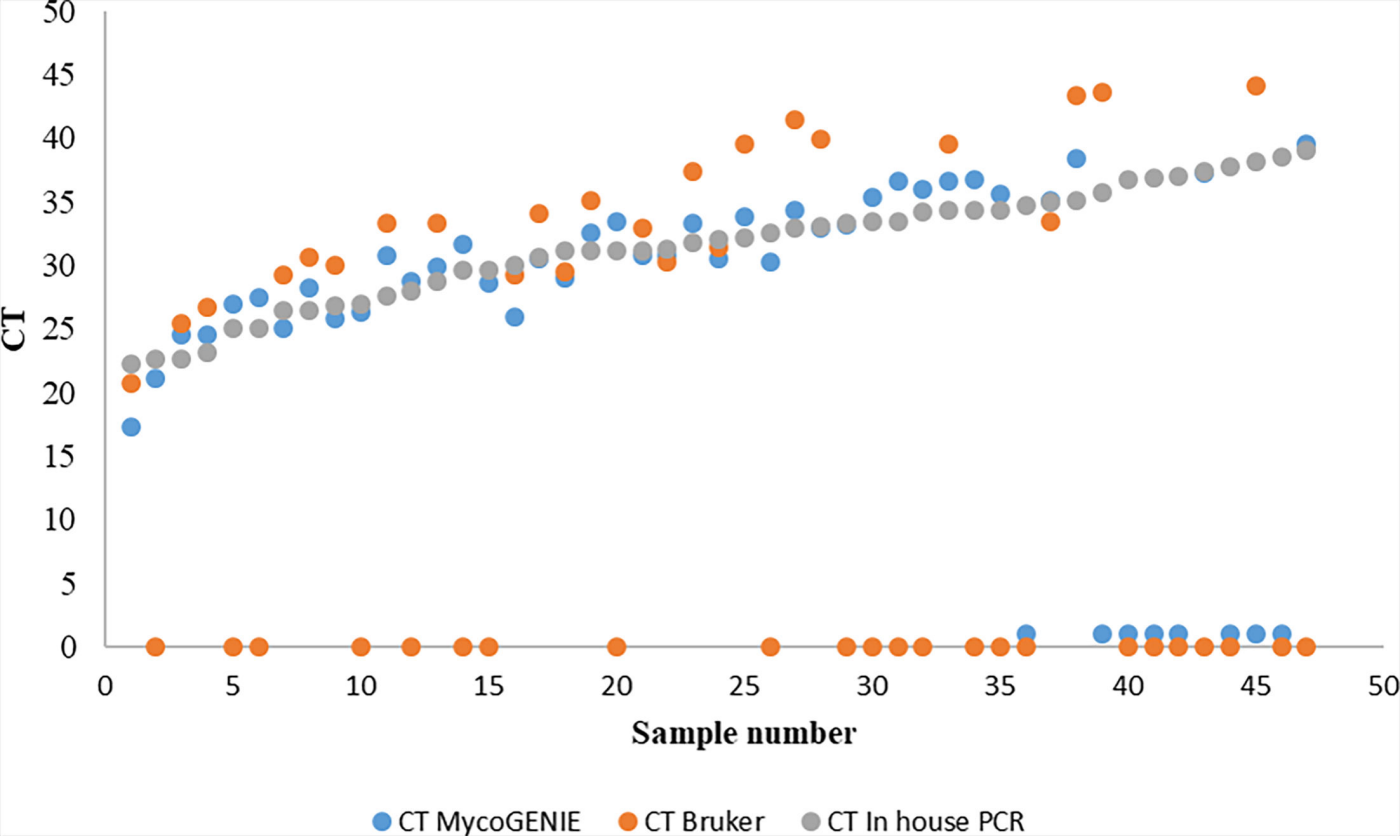
Comparative cycle threshold values obtained by the two PCR kits (Fungiplex, orange dots; MycoGENIE, blue dots) and the in-house Mucorales PCR (gray dots). The 47 clinical samples containing Mucorales DNA detected by the in-house PCR are sorted in ascending order of the CT value.

We compared the CT values obtained for each of the 53 samples using the three methods (Fig. 2). The 39 positive PCR results with MycoGENIE Kit showed CTs close to that of the in-house PCR with an average CT difference of 1.6 cycles (IQR, 0.1–5). For the 24 positive PCR results with Fungiplex, the difference in CT with that of the in-house PCR was larger, with an average of 4.1 cycles (IQR, 0.6–8.4).

The eight false negative results obtained by the MycoGENIE had high CTs of ≥35 by in-house PCR. For the Fungiplex Kit, only 7 out of the 23 negative PCR had high CTs of ≥35 by in-house PCR.

The agreement between the two kits has been calculated by the kappa statistic. The kappa value was 0.18, corresponding to a poor agreement.

### Performances to detect the different *Mucorales* species in clinical samples

**Fig 3:**
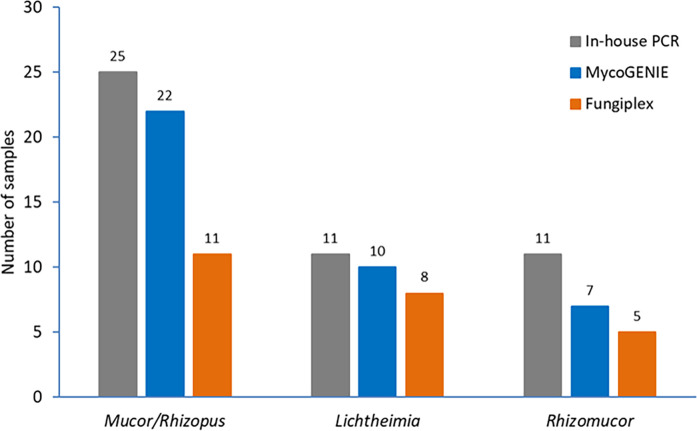
DNA detection by two PCR Mucorales kits compared to the result of the in-house PCR according to the genera of Mucorales. Each bar represents the number of positive samples obtained by the in-house PCR assay (gray bar), the Fungiplex Kit (orange bars), and the MycoGENIE Kit (blue bars).

In assessing the genera of Mucorales, the MycoGENIE Kit accurately detected 22 out of 25 samples (88%) positive for the genus *Mucor* spp./*Rhizopus* spp., 10 out of 11 samples (91%) positive for the genus *Lichtheimia* spp., and 7 out of 11 samples (64%) positive for the genus *Rhizomucor* spp. Meanwhile, the Fungiplex detected 11 out of 25 samples (44%) for the genus *Mucor* spp./*Rhizopus* spp., 8 out of 11 samples (73%) for the genus *Lichtheimia* spp., and 5 out of 11 samples (45%) for the genus *Rhizomucor* spp. (Fig. 3).

### Agreement between the MycoGENIE Kit and the in-house PCR

Agreement between the MycoGENIE Kit and the in-house PCR was evaluated using the data from all the 98 samples (Fig. 1, step 2). Among the 58 positive samples with the in-house Mucorales PCR, 49 samples were tested positive with the MycoGENIE Kit. Among the 40 negative control samples, 20 were positive for the detection of *Aspergillus fumigatus* DNA (Table S1), but all the 40 samples were detected negative for Mucorale DNA with the MycoGENIE Kit. The agreement between the MycoGENIE and in-house PCR was very good with a kappa value of 0.82.

**Fig 4:**
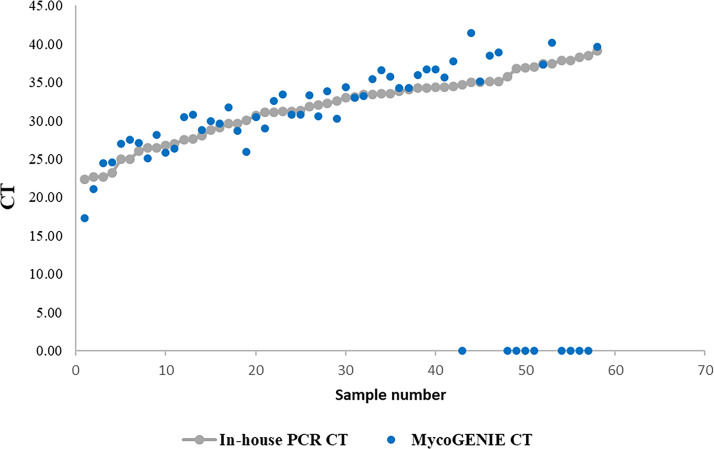
Comparative cycle threshold values obtained by the MycoGENIE PCR kit (blue dots) and the in-house PCR (gray dots). The 58 clinical samples containing Mucorales DNA detected by the in-house PCR are sorted in ascending order of the CT value.

Comparison of the CT values obtained with the MycoGENIE Kit and in-house PCR showed very low variability (Fig. 4). The average CT difference was 1.8 cycles (IQR, 0.1–5). Thus, the coefficient of variation is on average 3%, ranging from 0.1% to 11.2%. However, it should be noted that all six samples that tested positive by in-house PCR but negative by MycoGENIE Kit had CT values of ≥35 in the in-house PCR.

## DISCUSSION

Mucorales infections are distinguished by a notable release of Mucorales DNA, detectable in tissues and present in high amount in the bloodstream. DNA detection serves as a robust diagnostic marker for mucormycosis. Extensive research by L. Millon’s team has underscored the diagnostic and prognostic significance of DNA detection across various clinical forms—be it rhinocerebral, cutaneous, pulmonary, or disseminated—and irrespective of the genus involved (11, 12, 18). A recent clinical study led by this group advocates for the integration of Mucorales PCR as a fundamental diagnostic tool for these deadly infections (11). This recommendation stems from the utilization of a meticulously standardized and assessed in-house PCR assay, albeit not commercially available.

Concurrently, several manufacturers have developed kits for this purpose, yet only a handful have undergone comparative evaluation against the in-house PCR using identical clinical samples. Our study addresses this gap by assessing two CE-stamped kits against the in-house PCR on a substantial number of positive samples. The objective is to ascertain their potential as alternatives to the in-house PCR. Our findings reveal that one of the kits, namely the Fungiplex Kit, exhibits inadequate performance to supplant the in-house PCR. On the other hand, the second kit, MycoGENIE, demonstrates superior performance, although it still falls short of the accuracy achieved by the in-house PCR.

The diagnostic value of the MycoGENIE Kit has been previously evaluated in BAL (23) and serum (20), but in none of these studies, the analytic performances of this kit have been compared to the performance of the in-house PCR. It has to be noted that there is no published study on the evaluation of the Fungiplex Kit. Evaluation of the commercial kits is of prime importance to allow their integration in the workflow of the routine diagnosis in clinical microbiology laboratories.

The development of the molecular biological techniques is crucial to improve the early diagnosis of the mucormycosis infection to start specific treatment (antifungal and surgical treatment) without delay. It is well known that the delay in specific treatment is associated with a high mortality rate (7). It has to be noted that, up to now, guidelines for the diagnosis of mucormycosis only moderately recommend the use of PCR methods (9) due to the lack of standardization of these methods. This highlights the need for evaluation of standardized commercial kits. On the other hand, while mycological culture and histological examination are very specific, their sensitivity remains low for biological diagnosis. The difficulty of the mycological culture and the histological examination is related to the accessibility of the lesions and the processing time of the samples. On contrary, blood samples are easy to obtain and can be used for molecular testing in both diagnosis and screening strategies (11).

Our study evaluated the performance of two commercial kits for the detection of Mucorales DNA and showed the better performance of the MycoGENIE Kit in comparison to the Fungiplex Kit. Indeed, up to now, no study has compared the efficiency of these two kits. Although, according to the supplier’s recommendation, a lower volume of DNA extract was used for the reaction (10 µL for the MycoGENIE Kit and 5 µL for the Fungiplex Kit), this could not fully explain the differences in performances between the two kits. One of the drawbacks on the MycoGENIE is the inability to identify the genus and species of Mucorales because all the PCR targets were detected on the same channel. On contrary, the Fungiplex Kit allowed to identify a set of Mucorales species as three different channels is used to run the assay. Moreover, it should be noted that some species are not detected by Fungiplex Kit in comparison with MycoGENIE such as *M. irregularis* (formerly *Rh. variabilis*).

There are some limitations of the present study. The most important is the small sample size and the variety of samples included. However, this study encompassed the most prevalent samples utilized for the real-life diagnosis of mucormycosis. Notably, 54% of the samples were derived from blood, while 20% originated from the respiratory tract. Furthermore, the study incorporated invaluable invasive samples such as CSF, cutaneous biopsies, and sinus biopsies, which corresponded to the specific localization of the infection. Here, our study provides information on agreement between MycoGENIE and in-house PCR for DNA detection, but adequate evaluation of diagnostic accuracy needs to be tested in prospective real-world studies.

In conclusion, our study showed that the MycoGENIE Kit provides good performance but remains inferior to in-house PCR. Nevertheless, the MycoGENIE Kit does not provide any information regarding the genus responsible for infection. The current availability of reliable commercialized kits and their intrinsic performance validation opens the way to standardize molecular diagnosis of mucormycosis in routine clinical microbiology laboratories.

## SUPPLEMENTAL MATERIAL

Table S1 spectrum.03585-23-s0001.docx Mucorales DNA detection obtained by the ycoGENIE kit.

ASM does not own the copyrights to Supplemental Material that may be linked to, or accessed through, an article. The authors have granted ASM a non-exclusive, world-wide license to publish the Supplemental Material files. Please contact the corresponding author directly for reuse.
