# Supplementary material for: Agreement between two real-time commercial PCR kits and an in-house real-time PCR for diagnosis of mucormycosis
Source: Microbiol Spectr. 2024 Jun 25;12(8):e03585-23. doi: 10.1128/spectrum.03585-23 (PMC11302037; doi:10.1128/spectrum.03585-23)
Supplement: Table S1 — Mucorales DNA detection obtained by the ycoGENIE kit. [file spectrum.03585-23-s0001.docx]

**Suppl Table1:** Mucorales DNA detection obtained by the MycoGENIE® kit compared to in-house PCR. Muc + Afum +: samples positive for Mucorales and *Aspergillus fumigatus* using in-house PCR; Muc + Afum-: samples positive for Mucorales and negative for *Aspergillus fumigatus* using in-house PCR.

| Sample | Mucorales DNA detection by MycoGENIE®* | | Total |  | |  |
| --- | --- | --- | --- | --- | --- | --- |
|  | Positive | Negative |  | |  | |
| Muc + Afum + | 0 | 0 | 0 | |  | |
| Muc + Afum - | 49 | 9 | 58 | |  | |
| Muc - Afum + | 0 | 20 | 20 | |  | |
| Muc - Afum - | 0 | 20 | 20 | |  | |

*The detection of Aspergillus DNA by the kit was not evaluated
